# Monocytes as an early risk factor for acute graft-versus-host disease after allogeneic hematopoietic stem cell transplantation

**DOI:** 10.3389/fimmu.2024.1433091

**Published:** 2024-09-12

**Authors:** Huimin Sun, Linjie Wu, Xueying Zhao, Yingying Huo, Peiyuan Dong, Aiming Pang, Yawei Zheng, Yiwen Han, Shihui Ma, Erlie Jiang, Fang Dong, Tao Cheng, Sha Hao

**Affiliations:** ^1^ State Key Laboratory of Experimental Hematology, National Clinical Research Center for Blood Diseases, Haihe Laboratory of Cell Ecosystem, Institute of Hematology and Blood Diseases Hospital, Chinese Academy of Medical Sciences and Peking Union Medical College, Tianjin, China; ^2^ Department of Hematology, Beijing Chao-Yang Hospital, Capital Medical University, Beijing, China; ^3^ Tianjin Institutes of Health Science, Tianjin, China

**Keywords:** aGVHD, HSCT, monocytes, hematopoietic reconstruction, risk factor

## Abstract

Acute graft-versus-host disease (aGVHD) is a major complication after allogeneic hematopoietic stem cell transplantation (allo-HSCT) and contributes to high morbidity and mortality. However, our current understanding of the development and progression of aGVHD after allo-HSCT remains limited. To identify the potential biomarkers for the prevention and treatment of aGVHD during the early hematopoietic reconstruction after transplantation, we meticulously performed a comparative analysis of single-cell RNA sequencing data from post-transplant patients with or without aGVHD. Prior to the onset of aGVHD, monocytes in the peripheral blood of patients with aGVHD experienced a dramatic rise and activation on day 21 post-transplantation. This phenomenon is closely aligned with clinical cohort results obtained from blood routine examinations. Furthermore, *in vitro* co-culture experiments showed that peripheral blood monocytes extracted from patients with aGVHD approximately 21 days post-transplantation induced a significantly higher proliferation rate of allogeneic T cells compared to those from patients without aGVHD. Our study indicates that monocytes could be a crucial early clinical risk factor for the development of aGVHD, and this insight could potentially guide the timing of monitoring efforts, recommending assessments at the pivotal juncture of approximately day 21 post-transplantation, shedding fresh light on the significance of early hematopoietic regeneration in relation to the onset of aGVHD.

## Introduction

Despite the routine use of graft-versus-host disease (GVHD) prophylaxis, acute GVHD (aGVHD) still affects 30%–60% of patients receiving allogeneic hematopoietic stem cell transplantation (allo-HSCT) and is associated with poor clinical prognosis (1–3). In recent years, there has been an increase in the number of allo-HSCT procedures conducted annually due to technological advancements and the promotion of haploidentical allo-HSCT (4–6). It also indicates that there will be a sharp increase in the number of aGVHD patients. The development of aGVHD was initially observed as a secondary disease that appeared after the recovery from conditioning-induced toxicity in murine models of bone marrow transplantation (7). After that, Billingham formulated three conditions for the development of aGVHD: the graft comprises immunologically competent cells, the recipient expresses tissue antigens that are absent in the transplant donors, and the recipient is incapable of eradicating the transplanted cells (8). Additionally, the development of aGVHD can be conceptually separated into three phases: activation of antigen-presenting cells (APCs); donor T-cell activation, proliferation, differentiation, and migration; and target tissue damage (9).

Considering the high morbidity and mortality of aGVHD, precisely predicting the occurrence of aGVHD is of vital importance for early intervention. Current pretransplant clinical risk factors for aGVHD mainly include human leukocyte antigen (HLA) compatibility, the ages and genders of recipients and donors, and conditioning regimen intensity (10, 11). When aGVHD manifests clinically, specific biomarkers including tumor necrosis factor receptor 1 (TNFR1), interleukin-33 receptor (ST2), and regenerating islet-derived protein 3-alpha (REG3α) are found in elevated levels in blood plasma, and immune cell infiltration has been detected in affected target organs like the liver, gut, and skin (12–14). Using biomarkers or risk models to predict the risk of aGVHD onset in patients receiving allo-HSCT can assist in effective clinical intervention (15, 16). However, the risk factors for aGVHD during the early hematopoietic reconstruction require further elucidation.

During the initial stages of HSCT, hematopoietic stem and progenitor cells (HSPCs) could be regulated in several ways, including a range of inflammatory signals, which could alter their differentiation bias (17, 18). Taking advantage of the rapid development of single-cell RNA sequencing (scRNA-seq) technology, we dissected the reconstitution dynamics of transplanted HSPCs at single-cell resolution in both mice and humans in previous studies (19, 20). More importantly, we identified a cluster of neutrophil progenitors with immunoregulatory function in mobilized human grafts, which have the potential against the development of aGVHD. However, in the context of aGVHD, a deeper comprehension is necessary of the hematopoietic reconstitution dynamics and intricate regulatory mechanisms of transplanted human HSPCs.

This study involved a comparative analysis at the single-cell level for the early hematopoietic reconstitution dynamics in aplastic anemia (AA) patients with or without aGVHD after allogeneic granulocyte colony-stimulating factor (G-CSF)-mobilized peripheral blood stem cell transplantation (allo-PBSCT). We found that patients with aGVHD had an obvious increase and activation of monocytes in day 21 peripheral blood (PB) post-transplantation and verified this phenomenon with clinical cohort and *in vitro* co-culture experiments. Our findings introduce a new risk factor for early prognostication of aGVHD, and monocytes could potentially serve as an intervention target for aGVHD management following transplantation.

## Methods

### Sample collection

All blood samples of patients were obtained from the Blood Diseases Hospital, Chinese Academy of Medical Sciences in China, and were collected into ethylenediaminetetraacetic acid (EDTA) tubes. Peripheral blood mononuclear cells (PBMCs) were isolated by density gradient centrifugation using Ficoll-Paque™ Plus (Gibco, Grand Island, NY, USA). The cells were frozen in CellBanker (AMSBIO, Cambridge, MA, USA), a fetal bovine serum (FBS)-free cryoprotectant, and stored in liquid nitrogen until further use.

### Single-cell RNA sequencing and data preprocessing

We included scRNA-seq data of total nucleated cells (TNCs) of PB and bone marrow (BM) from three healthy controls (HCs) and six patients, as well as scRNA-seq data of TNCs from patient-paired G-CSF-mobilized peripheral blood (donor) in our published study (20) (**Table 1**), and followed previous preprocessing and quality control using scanpy pipeline (Version 1.9.3) (21). Next, we normalized count data using *scanpy.pp.normalize_total* function and performed logarithmically transformation for the following analysis.

**Table 1:** Clinical parameters and outcomes of six AA patients undergoing allo-PBSCT.

Case ID	Age (years) (P)	Sex (P/D)	Diagnosis	Type of conditioning regimen	HLA-matched	aGVHD prophylaxis	aGVHD onset time and grade
P1	25	F/F	SAA	RIC	8/10	CSA+MMF	No
P8	17	M/M	SAA	RIC	10/10	FK506+MTX	No
P9	35	M/F	VSAA	RIC	10/10	CSA+MTX	No
P6	52	F/M	SAA	RIC	6/10	CSA+MTX+MMF	d37; grade I
P7	21	F/F	VSAA	RIC	5/10	FK506+MTX+MMF	d21; grade II
P10	18	M/F	SAA	RIC	5/10	FK506+MTX+MMF	d31; grade III

P, patient; D, donor; M, male; F, female; AA, aplastic anemia; SAA, severity AA; VSAA, very SAA; HLA, human leukocyte antigen; aGVHD, acute graft-versus-host disease; RIC, reduced intensity conditioning regimen; CSA, cyclosporine; MMF, mycophenolate mofetil; MTX, methotrexate; FK506, tacrolimus.

### Batch effect correction and cell type annotation

Using *scanpy.pp.highly_variable_genes*, we identified 1,869 highly variable genes for the following analysis. For principal component analysis, we regressed out the total number of counts and the proportion of mitochondrial counts and used the harmony algorithm to correct batch effects (22). We generated a neighborhood graph using *scanpy.pp.neighbors* with “neighbors = 30, npcs = 10” for downstream Uniform Manifold Approximation and Projection (UMAP) visualization and clustering analysis. We performed unsupervised clustering using *scanpy.tl.leiden*, and we identified 19 clusters by setting “resolution = 0.8”. Next, we identified subclusters for monocytes, neutrophils, and lymphoid cells. We repeated the data integration and unsupervised clustering for monocytes and performed batch effect correction by harmony and bbknn (23). For T, B, NK, and neutrophils, we reran neighborhood graph computation and unsupervised clustering. We identified the cell types of subclusters according to the expression of marker genes.

### Differential gene expression analysis

Differentially expressed genes (DEGs) were detected using the *scanpy.tl.rank_genes_groups* function with the Wilcoxon rank-sum test. Genes with an absolute value of *log foldchange* more than 1 and adjusted *p*-value less than 0.05 were defined as DEGs.

### Gene set enrichment analysis

For functional annotation of DEGs, we performed gene set enrichment analysis by Metascape (24) (Version 3.5.2) and used terms in *GO Molecular Functions and GO Biological Processes*. The R package pheatmap (Version 1.0.12) was used for visualizing the gene expression and functional annotation results.

### Cell–cell communication analysis

CellChat (Version 1.6.1) (25) was used to assess the cell–cell interactions between monocytes and lymphoid cells. The normalized gene expression data and CellChat human database were taken as input. Genes that expressed more than 10% of the cells in one cluster and the ligand–receptor pairs with *p*-values less than 0.05 were considered significant interaction molecules among different cell types. Results were visualized using functions in CellChat.

### Calculation of the signature score

The signature scores for monocytes and T cells were calculated by *scanpy.tl.score_genes* with functional gene sets from published studies.

### Statistical analysis

FlowJo, version 10.8.1, was used for analysis of flow cytometry data. Statistical comparison was performed using R (Version 4.2.3). *p*-Values for the Mann–Whitney U test and Tukey–Kramer test were calculated using the “stats” package, and the significance was shown as *p < 0.05, **p < 0.01, ***p < 0.001, and **** *p* < 0.0001.

### Monocyte-allogeneic T-cell co-culture experiments

T cells were isolated from fresh PBMCs of healthy volunteers using human CD3 MicroBeads, following the manufacturer’s instructions (Miltenyi Biotec, Bergisch Gladbach, Germany). Similarly, monocytes were isolated from cryopreserved human PBMCs using human CD14 MicroBeads (Miltenyi Biotec). Isolated cells were confirmed to consist of >95% target cells by flow cytometry (BD Canto II flow cytometer, BD Biosciences, San Jose, CA, USA). T cells were labeled with 1 μL carboxyfluorescein succinimidyl ester (CFSE) (Invitrogen, Carlsbad, CA, USA) and were co-cultured with sorted monocytes in 96-well U-bottom plates at 37°C with 5% CO_2_ at a 4:1 ratio in RPMI 1640 medium supplemented with 1%100 IU/mL penicillin, 10 μg/mL streptomycin (Gibco), 1% 2 mM l-glutamine (Invitrogen), and 10% heat-inactivated FBS (Gibco) for 5–7 days. Then, cultured cells were harvested into a FACS tube; incubated with antibodies CD4, CD8, CD25, and CD69 (BioLegend, San Diego, CA, USA); and analyzed using a BD Canto II flow cytometer.

### Criteria of clinical cohorts

#### Inclusion criteria

Patients’ ages ranged from 15 to 60 years.
Patients were diagnosed with aplastic anemia or acute leukemia and underwent allo-PBSCT.
Patients underwent conditioning regimens before allo-PBSCT.
Patients complied with study procedures and follow-up.

#### Exclusion criteria

Patients had co-occurring chronic diseases such as hepatitis and diabetes mellitus.
Patients were diagnosed with acute myelomonocytic leukemia.
Patients with abnormal liver function or gastrointestinal complications required further diagnostic evaluation.
Patients relapsed during 60 days post-transplantation.

## Result

### The early hematopoietic reconstitution is altered in transplant patients with aGVHD

**Figure 1:**
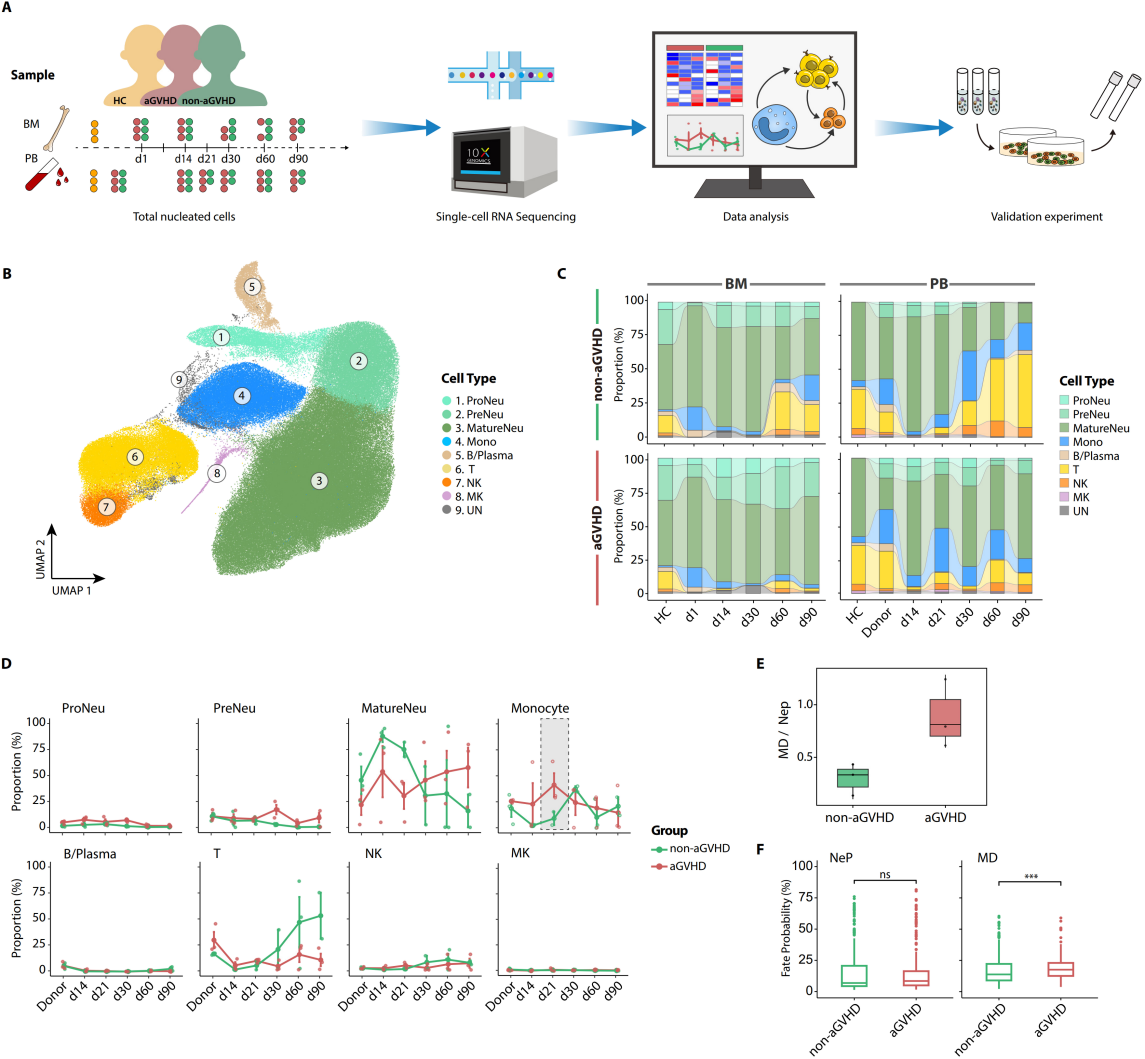
Comparison of hematopoietic reconstruction between patients with acute graft-versus-host disease (aGVHD) and those without aGVHD during early post-transplantation. **(A)** Overview of experimental design and data analysis. **(B)** Uniform Manifold Approximation and Projection (UMAP) visualization of total nucleated cells (TNCs) from healthy controls (HCs), granulocyte colony-stimulating factor (G-CSF)-mobilized peripheral blood (donors), and six patients. **(C)** The post-transplant cell compositions in patients with (bottom) or without aGVHD (upper) at multiple follow-up time points. **(D)** The dynamic proportion of TNC subsets between aGVHD and non-aGVHD groups at multiple follow-up time points. The monocyte proportion from d21 PB shows a sharp increase in aGVHD group. The line plots show the means ± SEM for the proportions of each cell type. **(E)** The ratio of monocyte/dendritic progenitors (MDs) to neutrophil progenitors (NePs) in d14 hematopoietic stem and progenitor cells (HSPCs) in bone marrow (BM). **(F)** Cell fate probabilities of hemopoietic stem cell multipotent (HSC/MPP), lymphoid-primed multi-potential progenitor (LMPP), and granulocyte-monocyte progenitor (GMP) in BM on d14 post-transplantation. *p*-Values were evaluated by the two-tailed Mann–Whitney U test. ns, not significant; ***p < 0.001.

To investigate the dynamics of early hematopoietic reconstruction in patients with aGVHD, we involved published scRNA-seq data of TNCs of PB and BM from three HCs and six patients, as well as scRNA-seq data of TNCs from patient-paired G-CSF-mobilized peripheral blood (donor). The case ID of six patients involved in the study corresponds one-to-one to the patients in our previous work (20). Patients included in this study were diagnosed as AA and underwent allo-PBSCT after BM conditioning. Three of them developed different grades of aGVHD and received surging immunosuppressive treatment, while the rest did not show any clinical manifestations of aGVHD within 6 months after allo-PBSCT (**Table 1**). The schematic workflow is depicted in **Figure 1A**, and the 10x Genomics platform was employed to generate single-cell transcriptome data of TNCs.

After rigorous quality control (**Supplementary Figure 1A**), 230,550 high-quality cells and 20,862 genes were obtained for subsequent analysis. All TNCs were visualized using UMAP and classified into eight major cell populations: Neutrophil progenitors (ProNeus), Neutrophil precursors (PreNeus), Mature neutrophils (MatureNeus), Monocytes (Monos), Megakaryocytes (MKs), B lymphocytes/Plasmas (B/Plasma), T lymphocytes, and Natural killer (NK) cells (**Figure 1B**; **Supplementary Figures 1B, C**).

Consistent with previous studies, neutrophils and monocytes emerged as the predominant cell populations during the first month after allo-PBSCT, while T cells remained largely absent until 30 to 60 days; the reconstruction state of T cells was greatly influenced by immunosuppressive therapy (20, 26), and these regulations of hematopoietic reconstruction showed consistency in both PB and BM (**Figure 1C**). Furthermore, the dynamics of hematopoietic reconstitution exhibited distinct characteristics and intriguing differences in PB compared to BM between the two groups. For example, the proportions of monocytes and MatureNeus in PB showed noteworthy differences between the aGVHD and non-aGVHD groups before the initial diagnosis of aGVHD (range from day 21 to day 37 after allo-PBSCT), and day 21 was a key time point of this hematopoietic reconstruction disparity. In the non-aGVHD group, MatureNeus had a higher proportion of PB within 21 days after allo-PBSCT. However, in the aGVHD group, the proportion of PB monocytes was higher within 21 days and reached the peak on day 21, and this enrichment of PB monocytes showed consistency among three aGVHD patient groups (**Figure 1D**; **Supplementary Figure 1D**). Therefore, we mainly focused on monocytes in PB on day 21 post-transplantation during subsequent analysis.

We further investigated possible explanations for the significant enrichment of day 21 PB monocytes in aGVHD patients. Monocytes from day 21 PB predominantly originated from the donors, with only 0.05% and 0.12% of monocytes being identified as recipient-derived cells by demuxlet (27) in the aGVHD and non-aGVHD groups, respectively (**Supplementary Figure 1E**). Considering that hematopoietic stem cells (HSCs), with the capacity of multilineage differentiation and self-renewal, are the origination of all reconstituted blood cell lineages (28), we introduced the scRNA-seq data of HSPCs in BM from HCs and patients 14 days post-transplantation involved in this study and compared the myeloid differentiation trajectory of HSPCs between the aGVHD and non-aGVHD groups (**Supplementary Figures 1F, G**). The ratio of monocyte/dendritic progenitors (MDs) to unipotent neutrophil progenitors (NePs) was elevated in patients with aGVHD than those without aGVHD (**Figure 1E**). The cell fate bias of multipotent and bipotent progenitors [estimated in a previous study (20)] on day 14 toward NePs exhibited no significant difference between the two groups while maintaining a pronounced inclination toward MDs in the aGVHD group than the non-aGVHD group (**Figure 1F**). Thus, the differentiation preference of HSPCs toward monocytes in the aGVHD group occurred prior to day 21, accounting for the abnormal regeneration of monocytes before the onset of aGVHD.

Taken together, the comparison analysis of scRNA-seq data systematically identified aGVHD-associated immune disturbances. A prominent enrichment of PB monocytes on day 21 after allo-PBSCT occurs in aGVHD patients, and this emergency monocytopoiesis stems from pre-existing differentiation bias of HSPCs.

### Prominent activation of enriched PB monocytes in aGVHD group on day 21 post-transplantation

**Figure 2:**
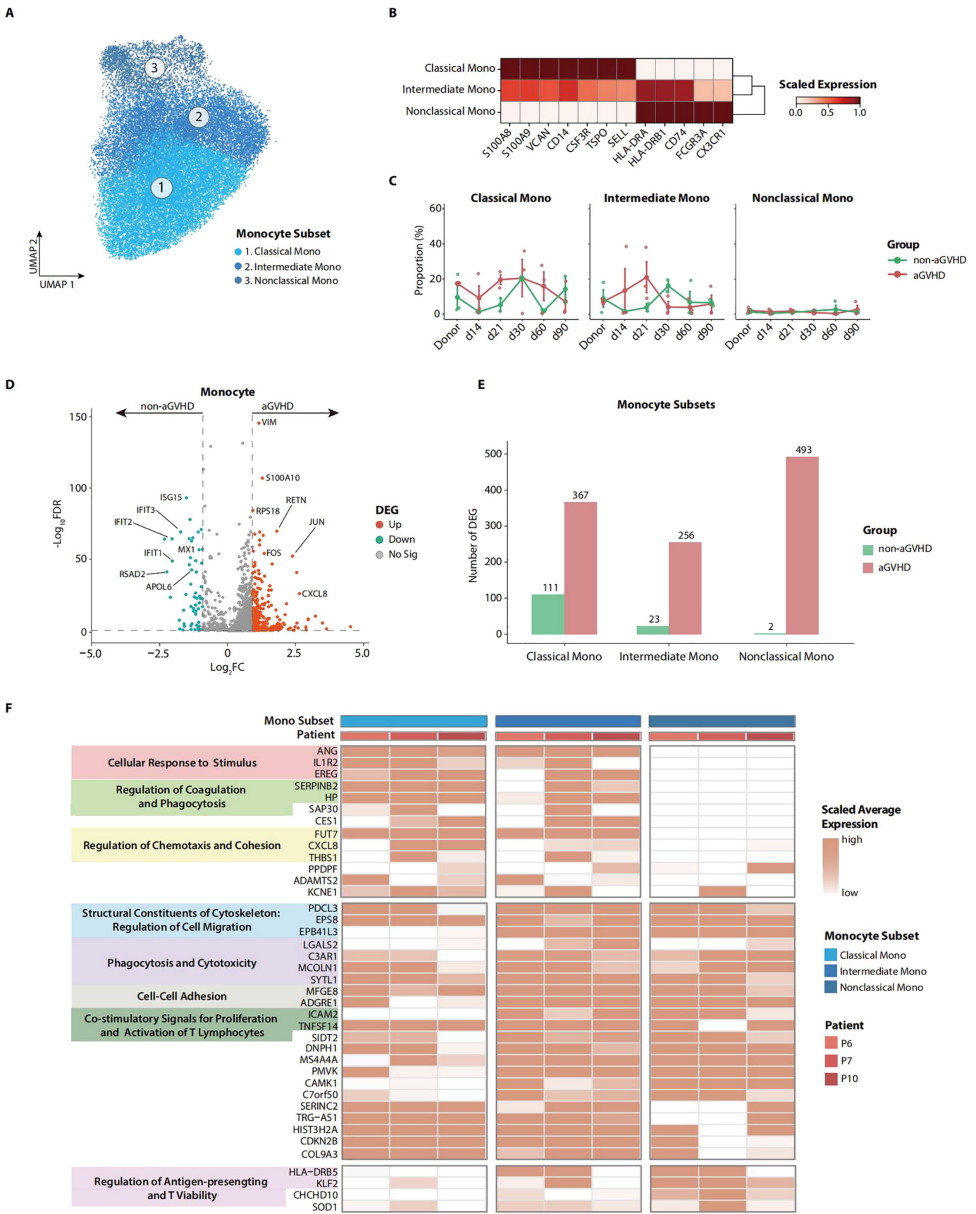
Transcriptome characteristics of peripheral blood (PB) monocytes on day 21 in patients with acute graft-versus-host disease (aGVHD). **(A)** Uniform Manifold Approximation and Projection (UMAP) of monocyte subsets. **(B)** Heatmap shows the marker genes for each monocyte subset. **(C)** The dynamic proportions of monocyte subsets in PB at multiple follow-up time points after allo-PBSCT. **(D)** Volcano plot depicts the differentially expressed genes (DEGs) in d21 PB monocytes between aGVHD and non-aGVHD groups. **(E)** Transcriptomic difference for each monocyte subset between aGVHD and non-aGVHD groups. The number of upregulated genes in the aGVHD group shows that the drastic transcriptomic variation occurred in all monocyte subsets. **(F)** Heatmap represents expression level of upregulated functional genes of each monocyte subset in d21 PB for aGVHD group. Annotations show functional attributes of genes in aGVHD group.

Considering the contribution of monocytes and monocyte-derived cells to the development of aGVHD (29, 30), we conducted further investigation into the functional variations of monocytes in patients with aGVHD. The heterogeneity of monocytes always corresponds to diverse functional specializations (31). To comprehensively characterize the functional variation of PB monocytes enriched on day 21, we compared the transcriptome profiles of monocyte subsets between the aGVHD and non-aGVHD groups. We defined three subsets in monocytes according to CD14 and CD16 expression: classical monocytes (CD14^++^CD16^−^), intermediate monocytes (CD14^++^CD16^+^), and non-classical monocytes (CD14^+^CD16^++^). Classical monocytes exhibited the highest expression levels of *S100A8* and *S100A9*, the proinflammatory mediator released by myeloid cells in many acute and chronic inflammatory disorders (32), while non-classical monocytes upregulated the expression of antigen presentation-associated genes, like *CD74*, *HLA-DRA*, and *HLA-DRB1* (33, 34) (**Figures 2A, B**). On day 21, the prominent enrichment of PB monocytes in the aGVHD group was primarily attributed to classical and intermediate monocytes, while the proportion of PB non-classical monocytes was comparable between the aGVHD and non-aGVHD groups (**Figure 2C**; **Supplementary Figure 2A**).

To further investigate the possible role of monocytes in the pathophysiology of aGVHD, we performed differential expression gene analysis for day 21 monocytes in PB. In the aGVHD group, monocytes showed higher proliferation and chemotaxis potential with the upregulation of genes like *FOS*, *JUN*, and *CXCL8* while downregulating interferon-associated genes like *ISG15* and *IFIT3* (**Figure 2D**). The aGVHD group demonstrated a significantly higher number of upregulated genes across each monocyte subset when contrasted with the non-aGVHD group. This observation highlighted the pronounced functional differences among the monocyte subsets between the two groups (**Figure 2E**; **Supplementary Figure 2B**). To further profile the functional characteristics of each monocyte subset, we annotated upregulated genes in monocyte subsets in the aGVHD group by gene ontology analysis. Classical monocytes showed activation in the regulation of phagocytosis, chemotaxis, and cohesion, while non-classical monocytes demonstrated enhanced capability of antigen-presenting and T-cell viability. The higher expression of *ICAM* and *TNFSF14*, the genes associated with co-stimulatory signals for T-cell proliferation and activation (35, 36), indicates that monocyte subsets may contribute to the proliferation and activation of T cells in the aGVHD group (**Figure 2F**).

We additionally explored the functional disparities of lymphocytes between the aGVHD and non-aGVHD groups. We defined elaborate subsets of T, B, and NK cells (**Supplementary Figures 2C, D**). By performing differential expression gene analysis for CD8 effector T and CD16 NK cells, we found that cytotoxicity-associated genes like *GZMB* and *KLF2* (37) were upregulated in the aGVHD group, implying the highly activated functional state of CD8 effector T and CD16 NK cells in aGVHD patients (**Supplementary Figure 2E**).

Collectively, the transcriptome profile of monocytes provides insights into the functional characteristics of monocytes from aGVHD patients. The overstated activation of monocytes may play an essential role in inducing T-cell activation and proliferation in the context of aGVHD.

### Enhanced cell–cell interactions between monocytes and cytotoxic cells occur in patients with aGVHD during the early hematopoietic reconstitution

**Figure 3:**
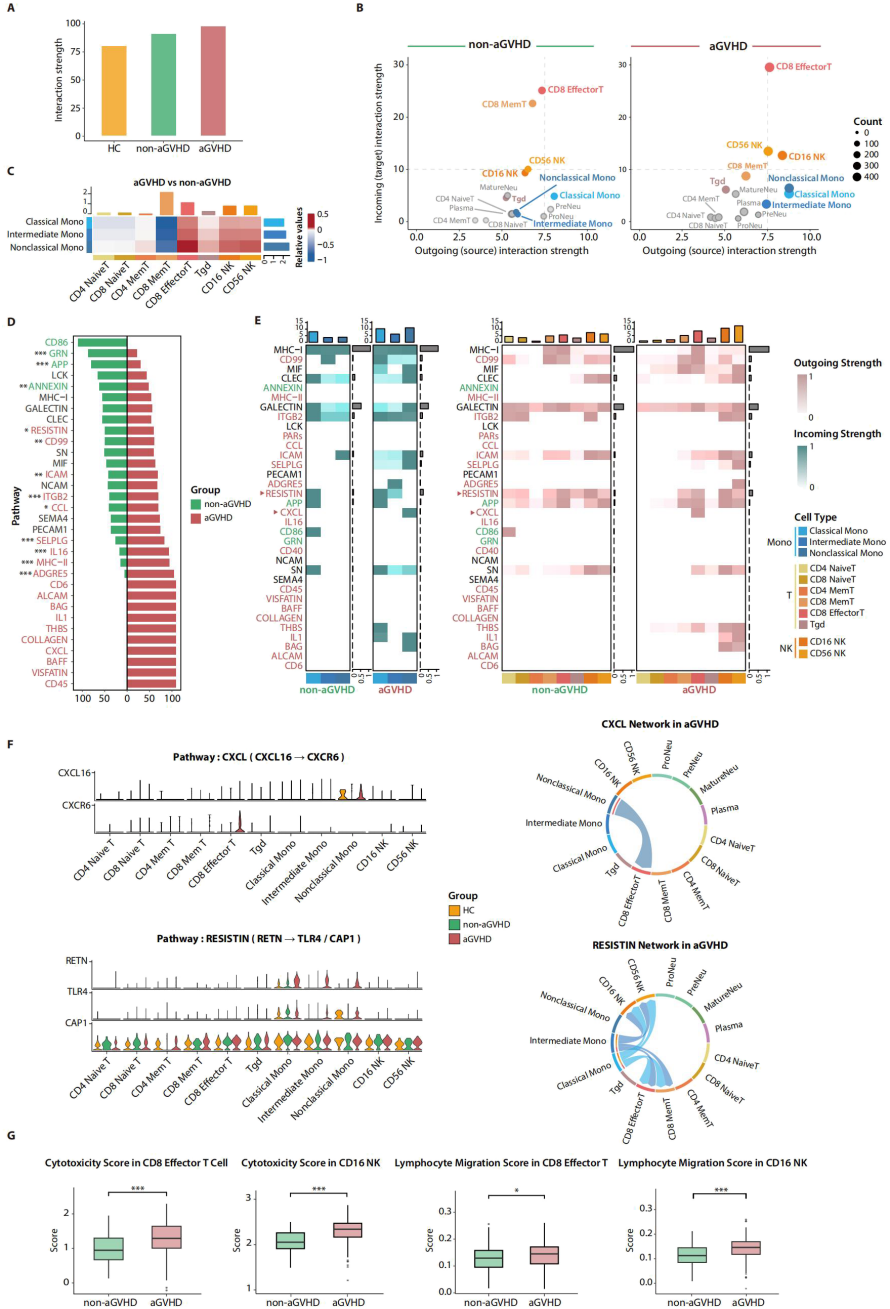
Cell–cell communication analysis for total nucleated cells (TNCs) in d21 peripheral blood (PB). **(A)** Barplot shows the strength of cell–cell interaction among healthy control (HC), acute graft-versus-host disease (aGVHD), and non-aGVHD groups. **(B)** Scatter plots show the outgoing and incoming strength for each cell type. Monocyte subsets, CD8 memory T, CD8 effector T, and NK subsets are the most variable cell types between aGVHD and non-aGVHD groups. CD4 Mem T, CD4 Memory T; CD8 Mem T, CD8 Memory T. **(C)** Relative value of interaction strength among the most variable cell types between aGVHD and non-aGVHD groups. The positive value (red) represents stronger interaction strength, and the negative value (blue) represents weaker interaction strength in aGVHD group. CD4 Mem T, CD4 Memory T; CD8 Mem T, CD8 Memory T. **(D)** Relative information flow for signaling pathways between aGVHD and non-aGVHD groups. The pathways with significantly enhanced information flow are highlighted using colors corresponding to their respective groups. *p*-Values were evaluated by the two-tailed Mann–Whitney U test. *p < 0.05, **p < 0.01, and ***p < 0.001. **(E)** Heatmap shows outgoing and incoming signal intensities of most variable cell types. CD4 Mem T, CD4 Memory T; CD8 Mem T, CD8 Memory T. **(F)** Gene expression levels of ligand–receptors of CXCL and RESISTIN pathways in HC, aGVHD, and non-aGVHD groups (left). Chord plots show these two pathways mediated the cell–cell interaction patterns in aGVHD group (right). CD4 Mem T, CD4 Memory T; CD8 Mem T, CD8 Memory T. **(G)** Module scores of functional gene sets in CD8 effector T and CD16 NK. *p*-Values were evaluated by the two-tailed Mann–Whitney U test. *p < 0.05, ***p < 0.001.

To further explore the effect of aGVHD-associated activation of monocytes on the cell–cell regulatory network, we performed cell–cell communication analysis for PB immune cells on day 21 using CellChat (25) software. Patients with aGVHD showed the highest interaction strength and numbers among the HC, aGVHD, and non-aGVHD groups, indicating enhanced cell–cell interactions (**Figure 3A**; **Supplementary Figure 3A**). Compared with the non-aGVHD group, the augmentation of interaction strength in aGVHD patients was mainly focused on monocytes, T cells, and NK cells. The enhanced interaction strength of three monocyte subsets was primarily attributed to the outgoing signals of interaction, emphasizing the pivotal role of monocytes in regulating other cell populations in the context of aGVHD. Correspondingly, the incoming signal was obviously strengthened for CD8 effector T, CD16 NK, and CD56 NK, supporting the potential activation of lymphocytes in the aGVHD group (**Figure 3B**). Furthermore, the aligned interactive interplay between three monocyte subsets and CD8 effector T, CD16 NK, and CD56 NK was also remarkably augmented in the aGVHD group, indicative of the stimulation role of monocytes on T cells and NK cells. In addition, the cell–cell communication between monocyte subsets and CD8 memory T was relatively weak in the aGVHD group, supporting the initiative role of cytotoxic lymphocytes including CD8 effector T and CD16 NK in inducing target damage of aGVHD (38) (**Figure 3C**; **Supplementary Figure 3B**).

To investigate the underlying mechanism of altered cell–cell interaction, we compared the level of involvement of all detected pathways between the aGVHD and non-aGVHD groups and revealed the different enrichment paradigms of signal pathways between the two groups. The upregulation of cytokine-associated pathways including RESISTIN, IL16, and IL1 pathways reflected that immune cells in aGVHD patients had a higher proinflammatory ability and stronger signal transmission, while the upregulated CCL, CXCL, and ITGB2 pathways indicated the enhanced ability of cell migration and cohesion of immune cells in the aGVHD group. Additionally, the signal pathways, including ICAM, MHC-II, and CD45, which play crucial roles in the co-stimulation of T cells, were found to be significantly enriched in the aGVHD group (**Figure 3D**), which was consistent with the observation in **Figure 2E**.

We subsequently investigated the pivotal role of enriched pathways in facilitating enhanced cell–cell interactions among monocytes, T cells, and NK cells in patients with aGVHD. The overall enrichment state of the same signal pathway remained consistent in both outgoing signal-originated monocyte subpopulations and incoming signal-received T and NK subsets, supporting that these pathways upregulated in the aGVHD group mediate the enhancement of cell–cell interactions among monocytes, T cells, and NK cells (**Figure 3E**). The CXCL pathway was specifically activated between non-classical monocytes and CD8 effector T cells in aGVHD patients, and the expression of the ligand–receptor pair genes *CXCL16* and *CXCR6* was respectively upregulated in non-classical monocytes and CD8 effector T cells. The CXCL pathway plays an important role in immune cell migration (39); thus, the activation of the CXCL pathway showed enhanced migration ability of CD8 effector T cells and non-classical monocytes in patients with aGVHD. Similarly, the RESISTIN pathway showed a preference for cell–cell interactions between classical, intermediate monocytes, and CD8 effector T cells and NK cells. In the aGVHD group, the expression of the ligand gene *RETN* was elevated on classical and intermediate monocytes, while the corresponding receptor gene *CAP1* was upregulated on CD8 effector T cells and NK cells (**Figures 3E, F**). These results demonstrate that monocytes may regulate the functional activity of T cells and NK cells by secreting immune effectors (40). In addition, even the identical pathways could exert different roles in mediating cell–cell interaction in conditions of aGVHD or no-aGVHD. The ITGB2 pathway mediated the interactions from monocytes to naive T cells and NK cells in the non-aGVHD group, whereas the interactions between monocytes and naive T cells were absent in the aGVHD group (**Supplementary Figure 3C**).

To elucidate the pivotal role of augmented cell–cell interactions within the aGVHD group in the pathogenesis of aGVHD, we calculated signature scores based on published gene sets to evaluate the killing and migration ability of T cells and NK cells (**Supplementary Table 1**). We found that both cytotoxicity and migration scores were significantly higher in the aGVHD group (**Figure 3G**). In addition, we scored the migration and cytokine secretion ability of monocytes. Monocytes in the aGVHD group also had higher scores of cytokine secretion and migration ability, which were especially noticeable in intermediate and non-classical monocytes (**Supplementary Figure 3D**).

In conclusion, we emphasized the enhanced regulatory network from monocytes to CD8 effector T cells and NK cells in the aGVHD group. These results further support that the day 21 monocytes in PB have the potential to induce the overstated activation and proliferation of T cells and NK cells in aGVHD patients.

### Insufficient immunosuppression may contribute to the development of aGVHD

Immunosuppression refers to the prevention or reduction of immune response, and insufficient immunosuppression can give rise to allograft rejection and recipient-specific antibody development (41). Myeloid-derived suppressor cells (MDSCs) are renowned for their roles in exerting anti-inflammatory and immunosuppressive effects, including two major subsets: polymorphonuclear (PMN)-MDSCs and monocytic (M)-MDSCs (42). In a previous study, we identified a cluster of neutrophil progenitors S100A^high^ Neu2 with the transcriptome characteristics of PMN-MDSCs, and these cells have the potential protection against the development of aGVHD (20). In this research, we found that PreNeus in PB had similar transcriptome characteristics with S100A^high^ Neu2. By scoring neutrophils with published gene sets of MDSCs, we found the signature scores were significantly higher in PreNeus than in other neutrophil subsets, showing that PreNeus may have immunosuppressive function (**Supplementary Figure 4A**; **Supplementary Table 1**).

On day 21 post-transplantation, significant transcriptomic disparities were observed between the two groups of PreNeus, with those from the non-aGVHD group exhibiting the most pronounced upregulation of DEGs (**Supplementary Figure 4B**). Volcano plot further demonstrated that PreNeus in the non-aGVHD group upregulated immunosuppression-associated genes such as *ARG1* and *IL4R* (43), suggesting that PreNeus in the non-aGVHD group are more likely to exert immunosuppressive function than those in the aGVHD group (**Supplementary Figure 4C**). In addition, we selected published gene sets related to immunosuppression functions to evaluate the impact of PreNeus cells on immune response. The module scores of three gene sets were all significantly higher in the non-aGVHD group, further supporting that PreNeus from non-aGVHD patients exhibits a greater potential for negative regulation of immune response (**Supplementary Figure 4D**; **Supplementary Table 1**). This phenomenon also suggests that immunosuppressive cells like PreNeus may be crucial for the development and progression of aGVHD.

### Functional validation and clinical value of the abnormal accumulation of day 21 PB monocytes in aGVHD monitoring during the early post-transplantation period

**Figure 4:**
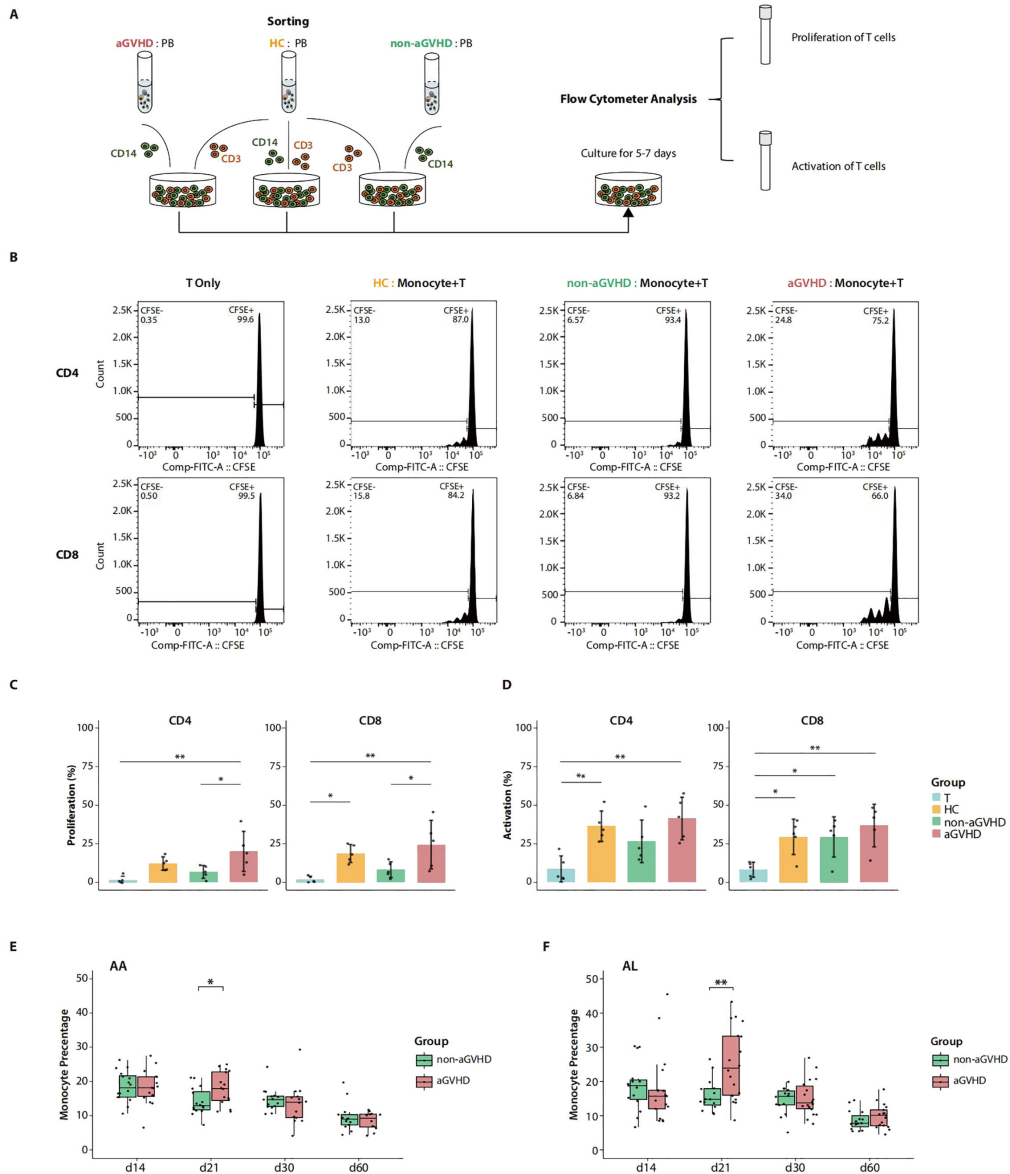
Co-culture experiments and clinical cohort. **(A)** Schematic overview of co-culture experiments of T cells and monocytes. **(B)** Flow cytometry graphs show the proliferation frequency of the allogeneic CD4^+^ T and CD8^+^ T cells estimated by carboxyfluorescein succinimidyl ester (CFSE) dilution after co-culture with monocytes. Monocytes were sorted from approximately day 21 peripheral blood (PB) of aplastic anemia (AA) patients undergoing allo-PBSCT with acute graft-versus-host disease (aGVHD) (aGVHD), without aGVHD (non-aGVHD), and PB of healthy controls (HCs). T cells cultured without monocytes (T Only) were used as the baseline control. **(C)** The summary of the proportion of T-cell proliferation (percentage of CFSE dilution) in co-culture experiments with five replications. *p*-Values were evaluated by the Tukey–Kramer test. *p < 0.05, **p < 0.01. **(D)** The summary of proportion of T-cell activation (percentage of CD69^+^/CD25^+^ T cells) in co-culture experiments with five replications. *p*-Values were evaluated by the Tukey–Kramer test. *p < 0.05, **p < 0.01. **(E)** Monocyte percentage from blood routine examination of AA patients with aGVHD (aGVHD group, n = 16) and without aGVHD (non-aGVHD group, n = 16) after allo-PBSCT. Each point represents the median value of monocyte percentages within 3 days around the corresponding time point. *p*-Values were evaluated by the two-tailed Mann–Whitney U test. *p < 0.05. **(F)** Monocyte percentage from blood routine examination of acute leukemia (AL) patients with aGVHD (aGVHD group, n = 18) and without aGVHD (non-aGVHD group, n = 15) after allogeneic peripheral blood stem cell transplant transplantation (allo-PBSCT). Each point represents the median value of monocyte percentages within 3 days around the corresponding time point. *p*-Values were evaluated by the two-tailed Mann–Whitney U test. **p < 0.01.

To validate the function of PB monocytes on day 21 in aGVHD patients, we collected PB samples from AA patients approximately day 21 after allo-PBSCT and isolated monocytes to co-culture with T cells from HCs for 5–7 days (**Figure 4A**). As expected, monocytes from aGVHD patients were noted to be more capable of inducing T-cell proliferation, while the T-cell activation showed no significance between the two groups (**Figures 4B–D**; **Supplementary Figure 5**). To further verify the clinical value of the enrichment of PB monocytes on day 21 in monitoring aGVHD onset, we collected the results of blood routine examination from 32 AA patients during 60 days after allo-PBSCT, including 16 aGVHD who were diagnosed as aGVHD during 21–100 days after transplantation and 16 non-GVHD patients who never manifested symptoms of aGVHD 120 days post-transplantation (**Supplementary Tables 2, 3**). By comparing the median cell percentage within 3 days for each post-transplantation time point, we observed similar monocyte enrichment on day 21 in aGVHD patients, which was consistent with the results concluded by transcriptome analysis (**Figure 4E**; **Supplementary Figure 6A**). In addition, we extended the observation of PB monocytes into acute leukemia (AL) patients receiving allo-PBSCT. For blood routine examination data from 33 AL patients after allo-PBSCT (18 aGVHD and 15 non-aGVHD patients), day 21 PB monocytes were also significantly enriched in the aGVHD group (**Figure 4F**; **Supplementary Figure 6B**; **Supplementary Tables 2, 3**). Moreover, the median time point for aGVHD onset was 36 days in the two clinical cohorts (**Supplementary Table 4**). These clinical data validated the phenomenon of the abnormal enrichment of PB monocytes on day 21 in aGVHD patients, supporting the potential of monocytes as an early risk factor to monitor the development of aGVHD for patients receiving allo-PBSCT clinically.

In this segment, we validated the functional superiority of day 21 PB monocytes from aGVHD patients in inducing the proliferation of T cells. In addition, aGVHD-associated aberrant accumulation of PB monocytes on day 21 after allo-PBSCT was also confirmed in clinical blood routine examination data from transplant patients with AA and AL, indicating the universality of the phenomenon. The overall findings suggest the promising potential of monocytes as an early-stage risk factor for the development of aGVHD.

## Discussion

HSCT is an established procedure for various disorders of the hematopoietic, immune, and metabolic systems. However, aGVHD remains the major complication of allo-HSCT and poses a threat to good prognosis. Here, our study provides new insights into the dynamics of the early hematopoietic reconstruction for patients with aGVHD after allo-PBSCT. We focused on immune cells from PB at different periods post-transplantation and found a significant increase in monocyte proportion on day 21 in the aGVHD group. The transcriptional profiling and *in vitro* co-culture experiments confirmed that day 21 PB monocytes isolated from aGVHD patients had a stronger ability to stimulate the proliferation of T lymphocytes than those of non-aGVHD patients. Furthermore, we verified our findings with clinical blood routine data, concluding that the aGVHD-associated enrichment of PB monocytes on day 21 post-transplantation could be generalized in patients undergoing allo-PBSCT, to some extent. Based on the dynamics of early hematopoietic reconstruction after transplantation, our findings provide new insights for early monitoring and therapeutic intervention of aGVHD.

Although the reconstitution dynamics of transplanted allogeneic HSPCs in both mice and humans have been described at single-cell resolution (19, 20, 44, 45), our understanding of the early hematopoietic reconstruction in the context of aGVHD is still severely limited. Previous studies have indicated that APCs can initialize and exacerbate GVHD in mice; however, the cell type has not been specified in humans, and associated GVHD prophylaxis regimens remain further to be developed (46–48). Clinical studies have revealed alterations in the proportions and phagocytic functions of monocyte subpopulations following complications after HSCT (49, 50). However, the biological characteristics and potential pathogenic role of monocytes in the development of aGVHD require further elucidation. Our research delineated the transcriptomic landscape of aGVHD progression during the initial phase of hematopoietic reconstitution and highlighted the abnormal accumulation and activation of day 21 PB monocytes before the occurrence of aGVHD. The abnormal accumulation of day 21 PB monocytes is close to being a universal phenomenon in patients undergoing allo-PBSCT and could be detected by clinical blood routine examination. In general, our study reveals that the abnormal accumulation of monocytes in PB on day 21 following allo-PBSCT is clinically feasible as a potential risk factor for aGVHD, and this groundbreaking discovery supports the advancement of aGVHD surveillance and intervention measures to coincide with this critical time point, specifically approximate day 21 post-transplantation.

Although we validated that monocytes in aGVHD patients have a stronger capability to induce the activation and proliferation of T cells compared with those in non-aGVHD patients, the spectrum of cytokines secreted by monocytes and T cells remains to be further explored. Of note, cytokines and related inflammatory pathways exert an essential role throughout the three phases of aGVHD pathophysiology. In the first phase, conditioning chemoradiotherapy or total body irradiation (TBI) traditionally provokes pathological tissue damage and promotes the release of proinflammatory cytokines [such as interleukin-1a, interleukin-33, tumor necrosis factor-α (TNF-α), and interleukin-1] as well as pathogen-associated molecular pattern (PAMP) molecules, which could significantly boost the antigen-presenting capacity of APCs (51–55). During the second phase of aGVHD pathophysiology, activated APCs induce the proliferation and activation of T cells by presenting alloantigens and secreting cytokines. Alloantigens are internalized, processed, and presented to T cells by APCs through the major histocompatibility complex (MHC)–peptide complex, which provides the first signal for T-cell activation. Interaction of costimulatory molecules on the APC and T-cell surface (including CD80/CD86-CD28 and CD40-CD40L) delivered a second costimulatory signal for T-cell activation. In addition, cytokines secreted by APCs are important components of the third signal of T-cell activation (56, 57). Systemic interleukin-6 (IL-6) concentration is elevated early after allogeneic transplant, and donor dendritic cell (DC)-derived IL-6 exerts a crucial regulatory role in the expansion and differentiation of T cells during aGVHD (58). Documented IL-6 signaling is critical to induce donor type-17 T (Th17) and type 22 T (Th22) cell differentiation after bone marrow transplantation (BMT) (59–61), and the anti-IL-6 receptor monoclonal antibody, tocilizumab, has been shown to effectively reduce the incidence of acute GVHD (62). Moreover, type I interferon (IFN) produced by putative DCs could enhance CD8^+^ T cell-mediated GVHD and graft-versus-leukemia (GVL), although protecting recipients from CD4^+^-mediated GVHD (63). Murine models also demonstrated that OX40, a molecule expressed on activated T cells and interacting with OX40L on activated APCs, stimulates effector T-cell proliferation. Furthermore, OX40 signaling in regulatory T cells (Tregs) disturbs their immunosuppressive effects (64, 65). In the third phase, differentiated effector cells, such as T cells and phagocytes—including monocytes and macrophages—secrete cytokines that contribute to the persistence and exacerbation of aGVHD. During aGVHD, Th17 and non-Th17 donor lineages are a primary source of granulocyte-macrophage colony-stimulating factor (GM-CSF), which can expand myeloid populations (66, 67). In addition, monocytes can achieve self-regulation through the secretion of GM-CFS during inflammatory response after HSCT (68). Collectively, these studies indicate that the combination of IL-6 and GM-CSF appears to establish a positive feedback mechanism, which significantly contributes to the progression of aGVHD.

Our study’s transcriptome analysis revealed that NK cells from patients with aGVHD exhibited heightened functional activation. A study demonstrated that NK cells migrate to GVHD target organs following a spatial and temporal distribution extremely similar to T cells after HSCT (69). Although there are still some controversies about the role of NK cells in aGVHD (70–72), much attention should be paid to NK cells because of the first donor-derived lymphocyte subset to recover (73) and their crucial role in GVL after HSCT for hematological malignancies (74). Kordelas L. et al. reported that the proportions of donor-derived NK cells expressing the activating receptor CD94/NKG2C were lower in recipients with GVHD compared with those without GVHD after HSCT. GVHD patients presented with a lower ratio of CD94/NKG2C to CD94/NKG2A on NK cells (75). Consistently, Ghadially and coworkers suggested that NK cells inhibited or promoted GVHD development by relying on different receptor expression profiles. NK cells with NKp46 receptor stimulation mediated the elimination of APCs, thereby reducing the incidence of GVHD, while the absence of NKp46 on donor NK cells results in DC-mediated increased stimulation of donor T, thereby facilitating GVHD development (76, 77). However, pro-inflammatory cytokines derived from NK cells contribute to GVHD development, which is well-established. Xun et al. showed that *in vitro* IL-2-activated human NK cells producing IFN-γ and TNF-α were able to induce aGVHD in a xenogeneic model (78). Furthermore, higher proportions of IFN-γ producing NK cells after HSCT were associated with an increased incidence of acute GVHD in humans (79). IFN-γ boosts the recognition of CD8 T cells for target cells and promotes the differentiation of CD4 T cells toward a T-helper type 1 (Th1) phenotype (80), which plays an important role in the pathophysiology of GVHD (81). Importantly, the cytokines (including type I IFN, interleukin-2, interleukin-18, and interleukin-15) secreted by dendritic cells, macrophages, monocytes can further promote NK-cell cytolysis and IFN-γ secretion (82). In short, NK cells can suppress GVHD by killing APCs, while NK cells can exacerbate GVHD due to enhanced NK-cell cytolysis or cytokine secretion facilitated by APCs. Further investigations are essential to elucidate the regulatory interactions between NK cells and other immune cells including T cells, monocytes, and neutrophils during aGVHD.

There are several intriguing perspectives that merit further studies. In patients with aGVHD, the differentiation bias of HSPCs toward monocytes occurs even prior to day 21 post-transplantation. Exploring the fundamental mechanisms behind the hematopoietic differentiation bias of HSPCs is anticipated to uncover potential targets for counteracting the abnormal accumulation of monocytes. In addition, there is uncertainty regarding the microenvironment in patients with aGVHD during the early hematopoietic reconstruction. The altered niche associated with aberrant monocyte accumulation could offer additional perspectives on the underlying pathogenic mechanisms. In addition, the regulatory role of immunosuppressive cells like PreNeus in the development of aGVHD remains under further investigation.

## Data Availability

The datasets presented in this study can be found in online repositories. The names of the repository/repositories and accession number(s) can be found below: GSE224714 (GEO) and HRA001359 (GSA; https://ngdc.cncb.ac.cn/gsa-human/browse/HRA001359).
