# Supplementary material for: Monocytes as an early risk factor for acute graft-versus-host disease after allogeneic hematopoietic stem cell transplantation
Source: Front Immunol. 2024 Sep 12;15:1433091. doi: 10.3389/fimmu.2024.1433091 (PMC11424452; doi:10.3389/fimmu.2024.1433091)
Supplement: Supplementary file 1 [file DataSheet1.pdf]

Supplementary Information

Monocytes as an early risk factor for acute graft-versus-host disease after allogeneic hematopoietic stem cell transplantation

Huimin Sun *et al.*

Supplementary materials in the PDF contains

Supplementary Figures：S1 to S6

Other Supplementary Material for this manuscript includes the following:

Supplementary Table：S1 to S3

Figure S1. Quality control and cell type annotation of single-cell RNA sequencing data.

(A) Boxplots show basic statics for quality control of total nucleated cells (TNCs). (B) Uniform Manifold Approximation and Projection (UMAP) visualization of cell subtype distribution of TNCs. CD4 Mem T: CD4 Memory T; CD8 Mem T: CD8 Memory T. (C) Dotplot shows marker genes for each cell cluster. (D) The dynamic proportion of PB TNCs for each patient. (E) The proportion of donor or patient monocytes in day 21 PB evaluated by demuxlet. (F) Diffusion map (left) visualization of HSPCs associated with myeloid differentiation from day 14 BM from post-transplantation patients and HCs. Dotplot (right) shows marker genes for each cell cluster. (G) Proportion of HSPCs associated with myeloid differentiation for HC and patients on day 14 BM post-transplantation. BM: bone marrow.

Figure S2. Comparison of PB TNCs on day 21 from aGVHD or non-aGVHD patients.

(A) The dynamic proportion of PB monocyte subsets on day 21 for each patient. (B) Venn plot shows the overlap of upregulated genes among monocyte subsets in aGVHD group. (C) UMAP of lymphocyte subsets. CD4 Mem T: CD4 Memory T; CD8 Mem T: CD8 Memory T. (D) Expression level of marker genes in each lymphocyte subset. (E) Volcano plots show the differential expression genes (DEGs) in CD8 effector T (left) and CD16 NK (right) from PB on day 21 between aGVHD and non-aGVHD groups.

Figure S3. Supplement results for cell-cell communication analysis for PB TNCs on day 21.

(A) The overall number of cell-cell interaction for HC, non-aGVHD and aGVHD

groups. (B) Interaction number (left) and interaction strength (right) among monocytes, Neus, T, Plasma, NK cells in aGVHD and non-aGVHD groups. (C) The ITGB2 pathway mediates interaction patterns and strength between monocytes and T, NK cells for non-aGVHD and aGVHD groups. CD4 Mem T: CD4 Memory T; CD8 Mem T: CD8 Memory T. (D) Signature scores for PB monocytes on day 21. *P* values were evaluated by the two-tailed Mann-Whitney U test. $^{*}P < 0.05$, $^{***}P < 0.001$.

Figure S4. PreNeu in day 21 PB from patients without aGVHD shows significantly immunosuppressive transcriptome characteristics.

(A) Signature scores among neutrophil subsets calculated by MDSC marker genes. *P* values were evaluated by the two-tailed Mann-Whitney U test. $^{***}P < 0.001$. (B) Number of DEGs in PB PreNeu on day 14, 21 and 30 between aGVHD and non-aGVHD groups after allo-PBSCT. (C) Volcano plot shows DEGs in PB PreNeu on day 21 between aGVHD and non-aGVHD groups. (D) Immune regulation scoring for PB PreNeu on day 21 from aGVHD and non-aGVHD patients by immunosuppression-related genes. *P* values were evaluated by the two-tailed Mann-Whitney U test. $^{***}P < 0.001$.

Figure S5. Flow cytometry results for co-culture experiments between monocytes and T cells.

(A) The flow cytometry graphs show activation frequency of allogeneic $CD4^{+}$ (percentage of $CD69^{+}CD4^{+}$ T cells and $CD25^{+}CD4^{+}$ T cells) and $CD8^{+}$ (percentage of $CD69^{+}CD8^{+}$ T cells and $CD25^{+}CD8^{+}$ T cells) T cells after co-culture with day 21 PB monocytes sorted from aGVHD or non-aGVHD patients undergoing allo-PBSCT and with monocytes from healthy controls and without monocytes, the latter were used as the baseline activation fraction of cells in each sample (aGVHD group, n=5; non-aGVHD group, n=5; HCs, n=5). (B) The summary of $CD4^{+}$ T cell activation frequency (percentage of $CD69^{+}CD4^{+}$ T cells and $CD25^{+}CD4^{+}$ T cells) from n = 5

experiments. P values were evaluated by Tukey-Kramer test. *$P < 0.05$, **$P < 0.01$. (C) The summary of $CD8^+$ T cell activation frequency (percentage of $CD69^+CD8^+$ T cells and $CD25^+CD8^+$ T cells) from n = 5 experiments. P values were evaluated by Tukey-Kramer test. *$P < 0.05$.

Figure S6. Cell percentages from blood routine examination for clinical cohorts.

(A) Lymphoid, neutrophil and promyelocyte percentages for 32 AA patients with or without aGVHD after allo-PBSCT (aGVHD group, n=16; non-aGVHD group, n=16). P values were evaluated by the two-tailed Mann-Whitney U test. *$P < 0.05$; **$P < 0.01$. (B) Lymphoid, neutrophil and promyelocyte percentages for 33 AL patients with or without aGVHD after allo-PBSCT (aGVHD group n=18; non-aGVHD group, n=15). P values were evaluated by the two-tailed Mann-Whitney U test. *$P < 0.05$.

Figure S1

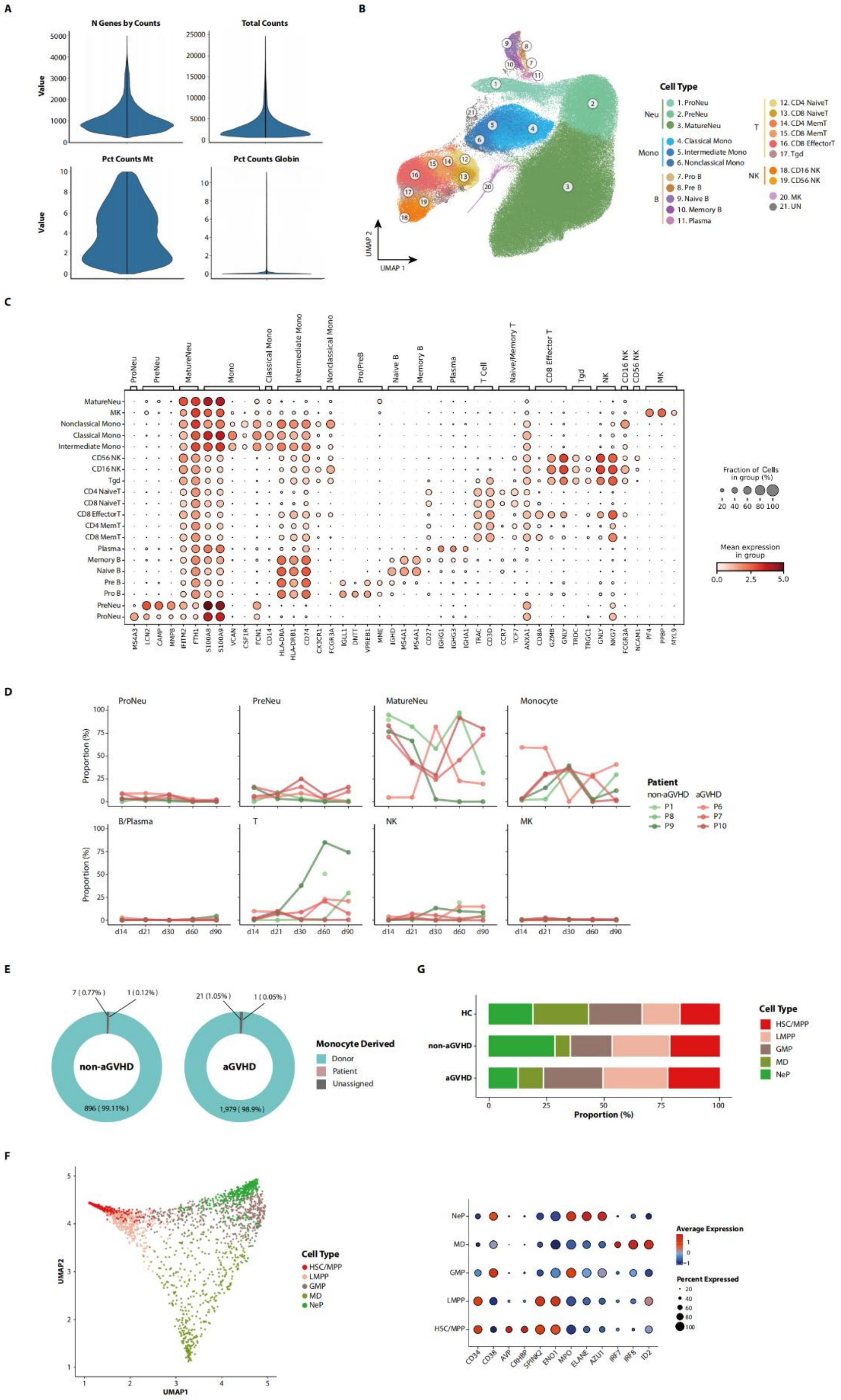

A
N Genes by Counts
Total Counts
Pct Counts Mt
Pct Counts Globin
Value
B
Cell Type
Neu
1. ProNeu
2. PreNeu
3. MatureNeu
Mono
4. Classical Mono
5. Intermediate Mono
6. Nonclassical Mono
B
7. Pro B
8. Pre B
9. Naive B
10. Memory B
11. Plasma
T
12. CD4 NaiveT
13. CD8 NaiveT
14. CD4 MemT
15. CD8 MemT
16. CD8 EffectorT
17. Tgd
NK
18. CD16 NK
19. CD56 NK
20. MK
21. UN
UMAP 1
UMAP 2
C
Fraction of Cells in group (%)
Mean expression in group
D
ProNeu
PreNeu
MatureNeu
Monocyte
B/Plasma
T
NK
MK
Proportion (%)
Patient
non-aGVHD
aGVHD
P1
P8
P9
P6
P7
P10
E
non-aGVHD
aGVHD
7 (0.77%)
1 (0.12%)
896 (99.11%)
21 (1.05%)
1 (0.05%)
1,979 (98.9%)
Monocyte Derived
Donor
Patient
Unassigned
G
HC
non-aGVHD
aGVHD
Proportion (%)
Cell Type
HSC/MPP
LMPP
GMP
MD
NeP
F
UMAP1
UMAP2
Average Expression
Percent Expressed

Figure S2.

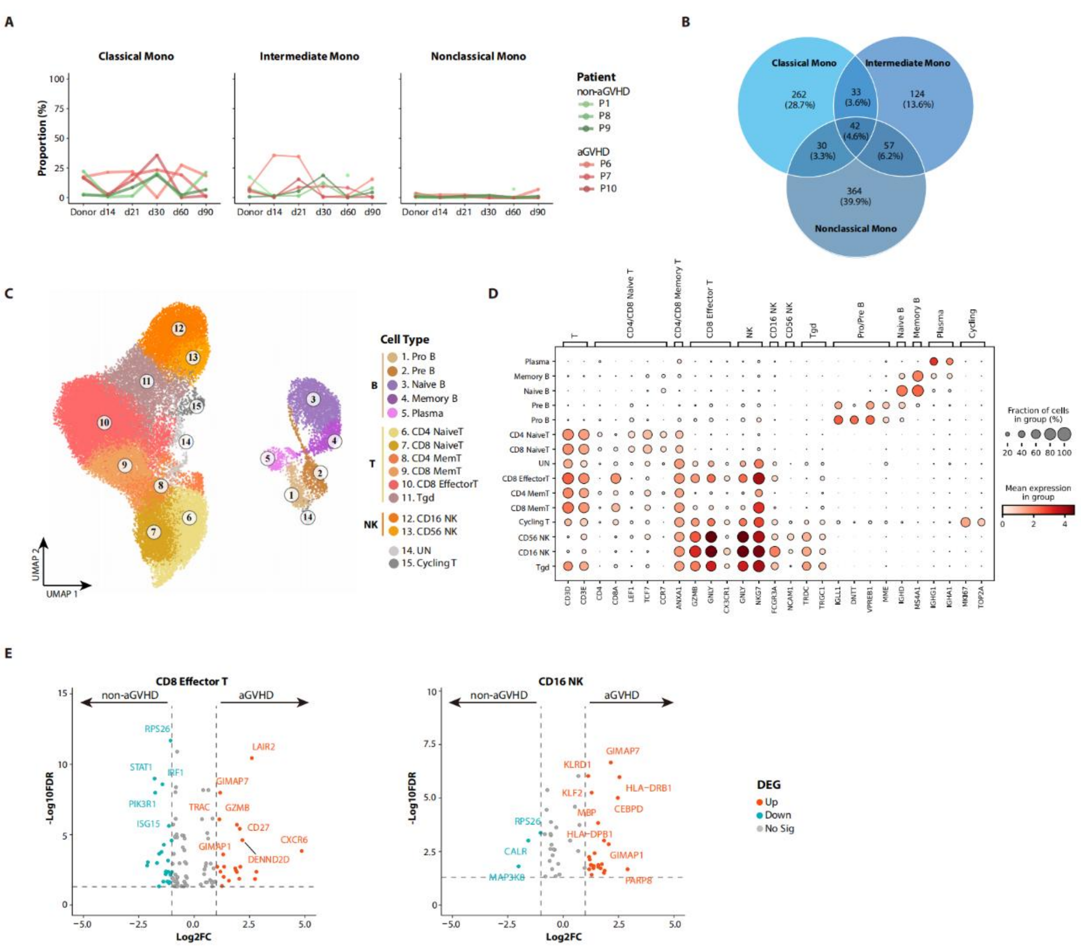

Figure S3.

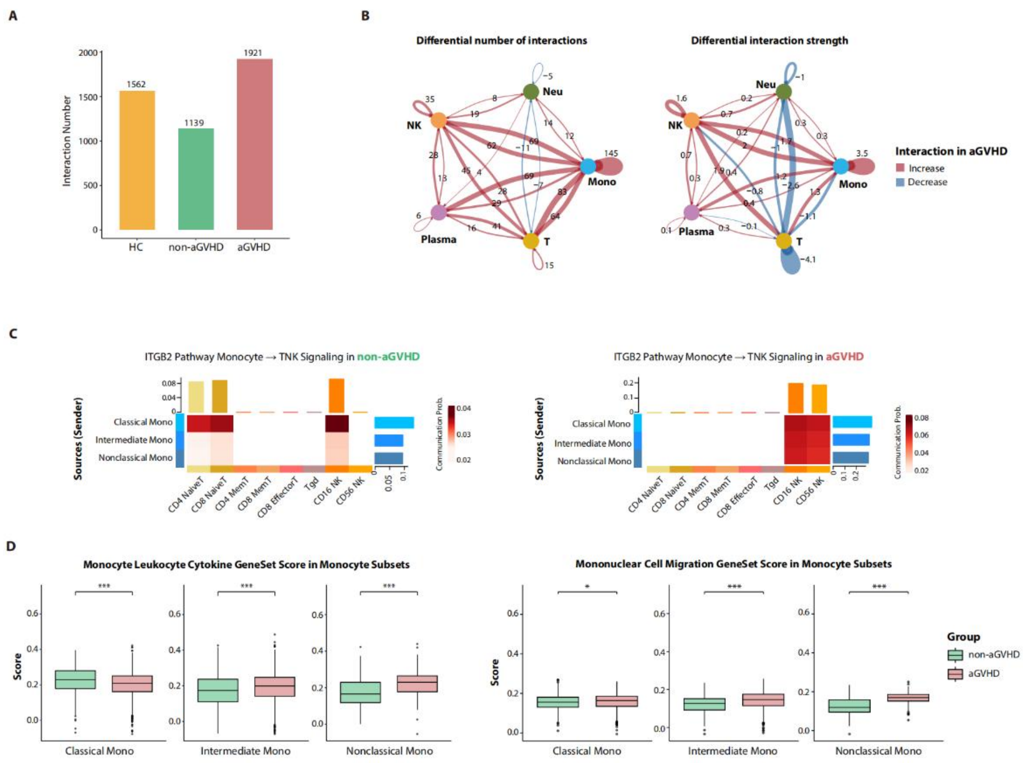
A
Interaction Number
1562
1139
1921
HC
non-aGVHD
aGVHD
B
Differential number of interactions
Differential interaction strength
Neu
NK
Mono
Plasma
T
Interaction in aGVHD
Increase
Decrease
C
ITGB2 Pathway Monocyte → TNK Signaling in non-aGVHD
ITGB2 Pathway Monocyte → TNK Signaling in aGVHD
Sources (Sender)
Classical Mono
Intermediate Mono
Nonclassical Mono
Communication Prob.
CD4 NaiveT
CD8 NaiveT
CD4 MemT
CD8 MemT
CD8 EffectorT
Tgd
CD16 NK
CD56 NK
D
Monocyte Leukocyte Cytokine GeneSet Score in Monocyte Subsets
Mononuclear Cell Migration GeneSet Score in Monocyte Subsets
Score
Classical Mono
Intermediate Mono
Nonclassical Mono
Group
non-aGVHD
aGVHD

Figure S4.

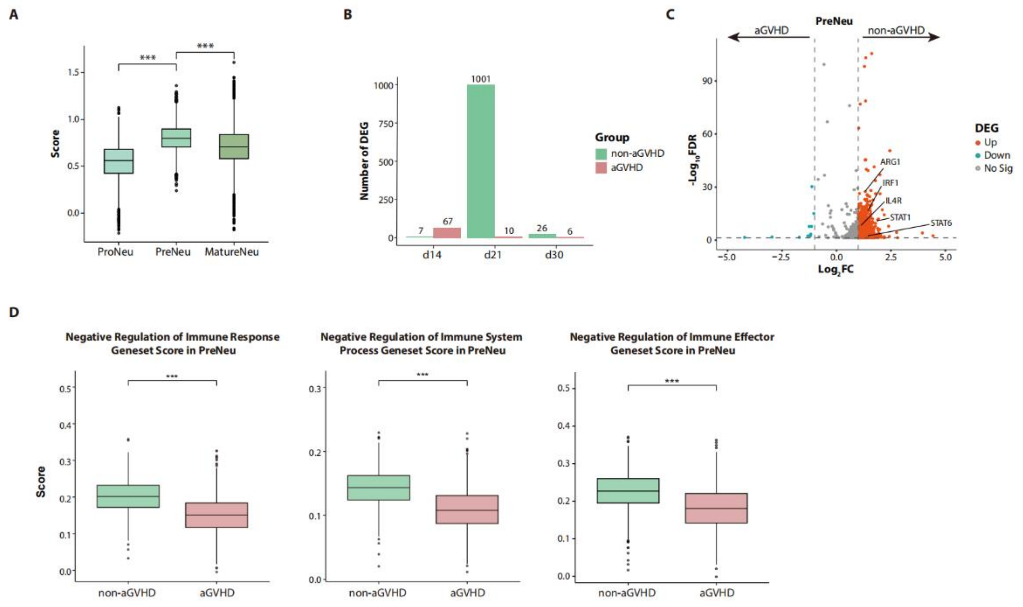
A
***
***
1.5
1.0
0.5
0.0
Score
ProNeu
PreNeu
MatureNeu
B
Number of DEG
1000
750
500
250
0
7
67
1001
10
26
6
d14
d21
d30
Group
non-aGVHD
aGVHD
C
PreNeu
aGVHD
non-aGVHD
90
60
30
0
-Log10FDR
ARG1
IRF1
IL4R
STAT1
STAT6
-5.0
-2.5
0.0
2.5
5.0
Log2FC
DEG
Up
Down
No Sig
D
Negative Regulation of Immune Response Geneset Score in PreNeu
Negative Regulation of Immune System Process Geneset Score in PreNeu
Negative Regulation of Immune Effector Geneset Score in PreNeu
***
Score
non-aGVHD
aGVHD

Figure S5.

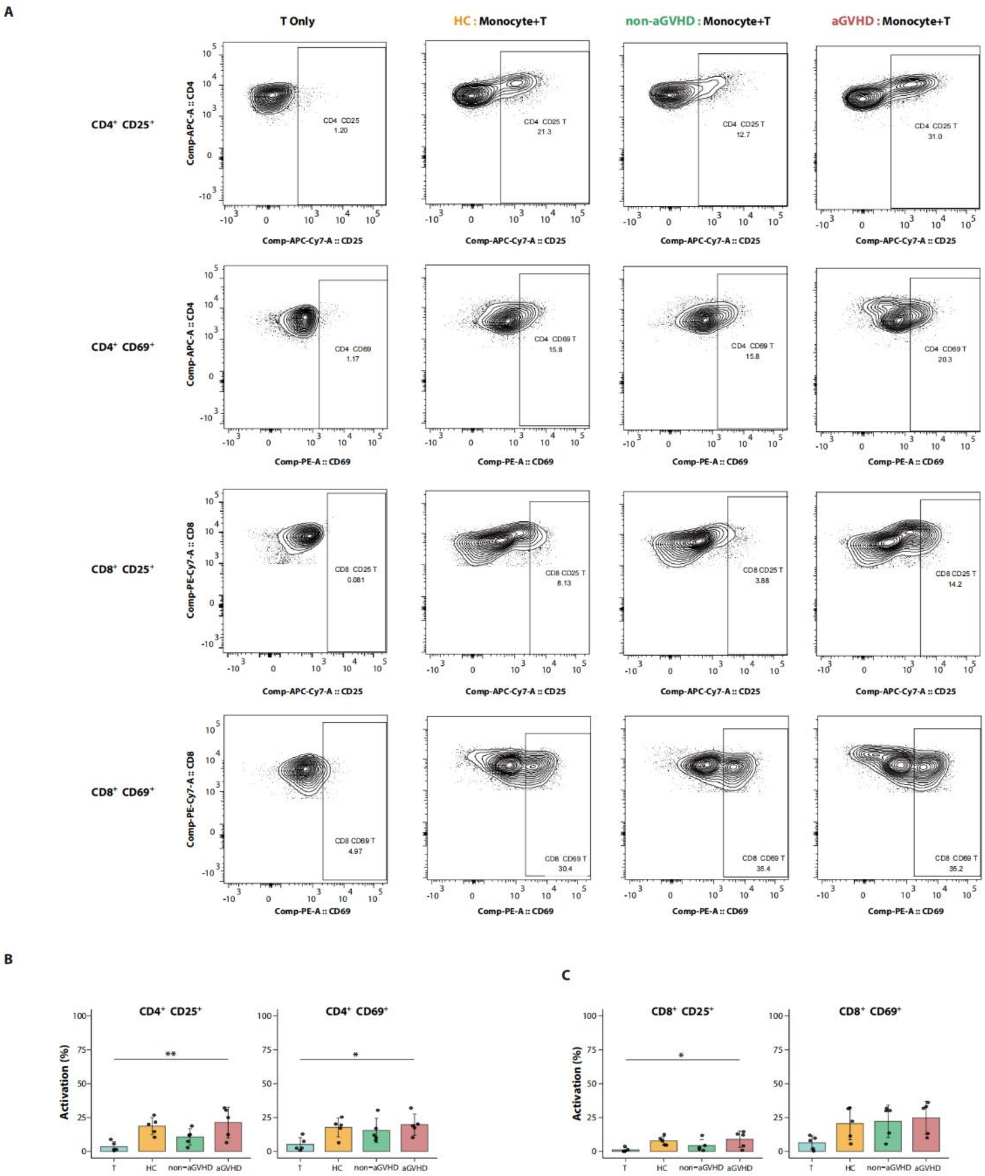
A
T Only
HC : Monocyte+T
non-aGVHD : Monocyte+T
aGVHD : Monocyte+T
CD4⁺ CD25⁺
CD4⁺ CD69⁺
CD8⁺ CD25⁺
CD8⁺ CD69⁺
Comp-APC-A :: CD4
Comp-PE-Cy7-A :: CD8
Comp-APC-Cy7-A :: CD25
Comp-PE-A :: CD69
CD4 CD25 1.20
CD4 CD25 T 21.3
CD4 CD25 T 12.7
CD4 CD25 T 31.0
CD4 CD69 1.17
CD4 CD69 T 15.8
CD4 CD69 T 15.8
CD4 CD69 T 20.3
CD8 CD25 T 0.081
CD8 CD25 T 8.13
CD8 CD25 T 3.88
CD8 CD25 T 14.2
CD8 CD69 T 4.97
CD8 CD69 T 30.4
CD8 CD69 T 35.4
CD8 CD69 T 35.2
B
C
Activation (%)
**
*
*
T
HC
non-aGVHD
aGVHD

Figure S6.

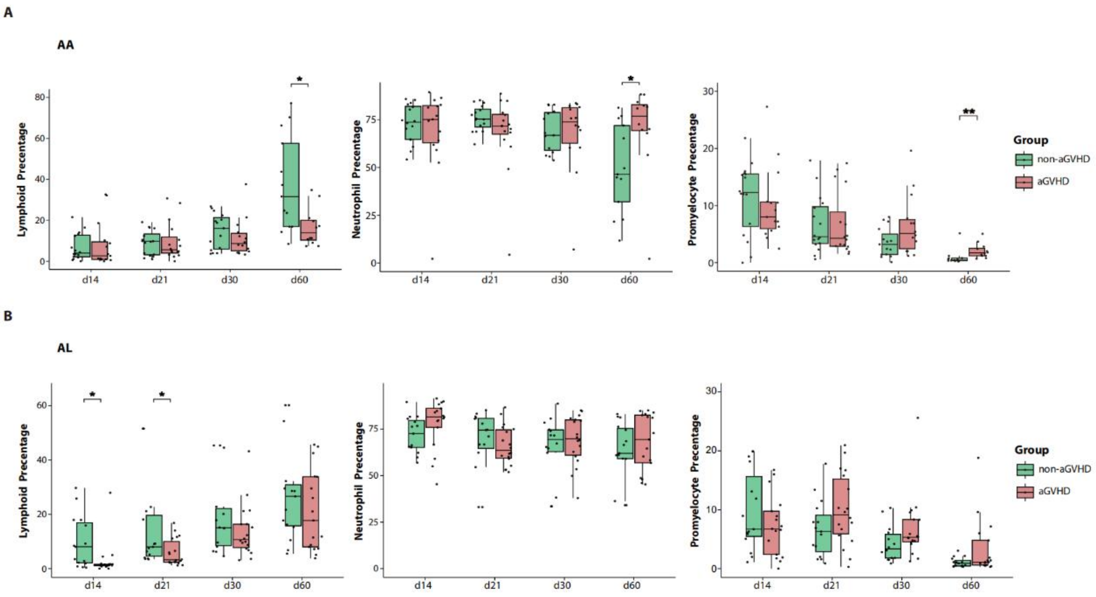
A
AA
Lymphoid Precentage
Neutrophil Precentage
Promyelocyte Precentage
d14
d21
d30
d60
Group
non-aGVHD
aGVHD
B
AL
