# Supplementary material for: Monocytes as an early risk factor for acute graft-versus-host disease after allogeneic hematopoietic stem cell transplantation
Source: Front Immunol. 2024 Sep 12;15:1433091. doi: 10.3389/fimmu.2024.1433091 (PMC11424452; doi:10.3389/fimmu.2024.1433091)
Supplement: Supplementary file 3 [file Table2.docx]

**Supplementary table S2: Clinical parameters and outcomes of 65 AA or AL patients undergoing allo-PBSCT.**

| **Case ID** | **Age(years)**  **(P)** | **Sex**  **(P/D)** | **Diagnosis** | **Type of conditioning regimen** | **HLA-matched** | **aGVHD prophylaxis** | **aGVHD onset time and grade** |
| --- | --- | --- | --- | --- | --- | --- | --- |
| AA1 | 25 | M/F | VSAA | RIC | 6/6 | CSA+MTX | No |
| AA2 | 19 | M/M | SAA | RIC | 5/10 | CSA+MTX+MMF | d34, grade？ |
| AA3 | 38 | M/F | SAA | RIC | 10/10 | CSA+MTX | No |
| AA4 | 34 | M/M | SAA | RIC | 10/10 | CSA+MTX | d60; grade II |
| AA5 | 18 | M/M | SAA | RIC | 10/10 | FK506+MTX | No |
| AA6 | 50 | F/M | SAA | RIC | 10/10 | CSA+MTX | No |
| AA7 | 18 | M/F | SAA | RIC | 5/10 | FK506+MTX+MMF | d31; grade Ⅳ |
| AA8 | 52 | F/M | SAA | RIC | 6/10 | CSA+MTX+MMF | d37; grade Ⅰ |
| AA9 | 21 | F/F | VSAA | RIC | 5/10 | FK506+MTX+MMF | d20; grade II |
| AA10 | 32 | M/M | AA | RIC | 5/10 | FK506+MTX+MMF | No |
| AA11 | 35 | M/F | VSAA | RIC | 10/10 | CSA+MTX | No |
| AA12 | 18 | M/F | SAA | RIC | 10/10 | CSA+MTX | No |
| AA13 | 15 | F/F | SAA | RIC | 10/10 | CSA+MTX+MMF | No |
| AA14 | 25 | M/M | FA | RIC | 5/10 | CSA+MTX+MMF | d21; grade? |
| AA15 | 19 | M/M | SAA | RIC | 10/10 | CSA+MTX | No |
| AA16 | 41 | F/M | VSAA | RIC | 10/10 | CSA+MTX | No |
| AA17 | 17 | M/F | AA | RIC | 10/10 | CSA+MTX | No |
| AA18 | 17 | M/M | VSAA | RIC | 5/10 | CSA+MTX+MMF | d35; grade Ⅲ |
| AA19 | 39 | M/F | SAA | RIC | 10/10 | CSA+MTX | d48; grade Ⅲ |
| AA20 | 21 | F/M | AA | RIC | 7/12 | MTX | d32; grade Ⅱ |
| AA21 | 36 | M/F | VSAA | RIC | 10/10 | CSA+MTX | d48; grade Ⅱ |
| AA22 | 19 | M/F | SAA | RIC | 10/10 | FK506+MTX | No |
| AA23 | 15 | F/M | SAA | RIC | 5/10 | CSA+MTX+MMF | d75; grade? |
| AA24 | 15 | M/F | AA | RIC | 12/12 | CSA+MTX | d50; grade Ⅱ |
| AA25 | 22 | F/M | SAA | RIC | 12/12 | CSA+MTX | No |
| AA26 | 27 | M/M | VSAA | RIC | 12/12 | CSA+MTX | d39; grade? |
| AA27 | 37 | M/M | SAA | RIC | 12/12 | MTX | d19; grade? |
| AA28 | 15 | M/M | VSAA | RIC | 8/12 | MTX | d24; grade Ⅳ |
| AA29 | 44 | M/M | VSAA | RIC | 12/12 | CSA+MTX | No |
| AA30 | 22 | M/F | SAA | RIC | 12/12 | CSA+MTX | No |
| AA31 | 37 | F/F | SAA | RIC | 12/12 | CSA+MTX | d54; grade Ⅱ |
| AA32 | 29 | F/M | VSAA | RIC | 7/12 | CSA+MTX+MMF | No |
| AL1 | 27 | F/F | ALL | MIC | 5/10 | CSA+MTX+MMF | d48; grade Ⅰ |
| AL2 | 34 | F/M | MAL | MIC | 6/12 | FK506+MTX+MMF | d90; grade? |
| AL3 | 55 | M/F | AML | MIC | 10/10 | FK506+MTX | No |
| AL4 | 34 | M/F | ALL | MIC | 12/12 | CSA+MTX+MMF | No |
| AL5 | 43 | M/M | ALL | MIC | 12/12 | MTX | d39; grade? |
| AL6 | 34 | F/M | MAL | MIC | 5/10 | CSA+MTX+MMF | d44; grade Ⅰ |
| AL7 | 29 | M/M | AML | MIC | 6/12 | CSA+MTX+MMF | d30; grade? |
| AL8 | 31 | F/F | AML | MIC | 6/12 | CSA+MTX+MMF | d30; grade? |
| AL9 | 36 | F/M | AML | MIC | 6/12 | CSA+MTX+MMF | d27; grade Ⅱ |
| AL10 | 31 | F/M | AML | MIC | 6/12 | CSA+MTX+MMF | d35; grade Ⅲ |
| AL11 | 32 | F/M | AML | MIC | 10/10 | FK506+MTX | No |
| AL12 | 40 | M/M | AML | MIC | 6/12 | CSA+MTX+MMF | d14; grade Ⅱ |
| AL13 | 42 | F/M | ALL | MIC | 6/12 | CSA+MTX+MMF | No |
| AL14 | 41 | F/M | ALL | MIC | 12/12 | MTX | d55; grade Ⅲ |
| AL15 | 47 | F/F | AML | MIC | 12/12 | FK506+MTX | No |
| AL16 | 48 | F/F | ALL | MIC | 10/12 | CSA+MTX+MMF | No |
| AL17 | 27 | M/M | ALL | MIC | 6/12 | CSA+MTX+MMF | d27; grade Ⅰ |
| AL18 | 55 | F/M | AML | MIC | 8/12 | CSA+MTX+MMF | d57; grade? |
| AL19 | 42 | M/F | AML | MIC | 7/12 | CSA+MTX | No |
| AL20 | 21 | F/F | ALL | MIC | 6/12 | FK506+MTX+MMF | d21; grade? |
| AL21 | 38 | F/F | MAL | MIC | 6/12 | CSA+MTX+MMF | No |
| AL22 | 35 | M/F | ALL | MIC | 12/12 | CSA+MTX | No |
| AL23 | 49 | M/M | AML | MIC | 7/10 | CSA+MTX+MMF | d39; grade Ⅱ |
| AL24 | 15 | M/M | AML | MIC | 6/12 | CSA+MTX+MMF | No |
| AL25 | 35 | M/F | ALL | MIC | 12/12 | CSA+MTX | No |
| AL26 | 50 | M/M | AML | MIC | 6/12 | FK506+MTX+MMF | No |
| AL27 | 49 | F/M | AML | MIC | 10/10 | CSA+MTX | No |
| AL28 | 20 | M/M | ALL | MIC | 6/12 | FK506+MTX+MMF | d26; grade Ⅱ |
| AL29 | 17 | M/F | AML | MIC | 6/10 | CSA+MTX+MMF | d100; grade? |
| AL30 | 48 | M/F | AML | MIC | 10/10 | CSA+MTX | d87; grade Ⅲ |
| AL31 | 50 | M/M | AML | MIC | 10/10 | FK506+MTX | No |
| AL32 | 51 | F/M | AML | MIC | 6/10 | FK506+MTX+MMF | d34; grade Ⅱ |
| AL33 | 37 | M/F | AML | MIC | 7/12 | CSA+MTX+MMF | No |

P: patient; D: donor; M: male; F: female; AA: aplastic anemia; SAA: severity AA; VSAA: very SAA; FA: fanconi anemia; HLA: Human leukocyte antigen; aGVHD: acute graft-versus-host-disease; AL: acute leukemia; AML: acute myelogenous leukemia; ALL: acute lymphoblastic leukemia; MAL: mixed acute leukemia; RIC: reduced intensity conditioning regimen; MIC: myeloablative conditioning regimen; CSA: cyclosporine; MMF: mycophenolate mofetil; MTX: methotrexate; FK506: tacrolimus
